# Burden of Pneumococcal Disease in Northern Togo before the Introduction of Pneumococcal Conjugate Vaccine

**DOI:** 10.1371/journal.pone.0170412

**Published:** 2017-01-23

**Authors:** Jennifer C. Moïsi, Makawa-Sy Makawa, Haoua Tall, Kodjo Agbenoko, Berthe-Marie Njanpop-Lafourcade, Stanislas Tamekloe, Moussa Amidou, Judith E. Mueller, Bradford D. Gessner

**Affiliations:** 1 Agence de Médecine Préventive, Paris, France; 2 Ministry of Health, Lomé and Dapaong, Togo; 3 Agence de Médecine Préventive, Ouagadougou, Burkina Faso; 4 Ecole des Hautes Etudes en Santé Publique (Sorbonne Paris Cité), Paris, France; 5 Unité Epidémiologie des Maladies Emergentes, Institut Pasteur, Paris, France; Public Health England, UNITED KINGDOM

## Abstract

**Background:**

*S*. *pneumoniae* is a leading cause of meningitis morbidity and mortality in the African meningitis belt, but little is known of its contribution to the burden of pneumonia in the region. We aimed to estimate the incidence of pneumococcal disease in children and adults in northern Togo, before the introduction of pneumococcal conjugate vaccine (PCV).

**Methods and findings:**

From May 1st 2010 to April 30th 2013, we systematically enrolled all hospitalized patients meeting a case definition of suspected meningitis or clinical pneumonia, residing in Tone or Cinkasse districts, northern Togo and providing informed consent. We collected clinical data and tested biological specimens according to standardized procedures, including bacteriology and PCR testing of cerebro-spinal fluid for meningitis patients and blood cultures and whole blood lytA PCR for pneumonia patients. Chest X-rays (CXR) were interpreted using the WHO methodology. We included 404 patients with meningitis (104 <5 years of age) and 1550 with pneumonia (251 <5 years) over the study period. Of these, 78 (19%) had pneumococcal meningitis (13 <5 years), 574 (37%) had radiologically-confirmed pneumonia (83 <5 years) and 73 (5%) had culture-confirmed pneumococcal pneumonia (2 <5 years). PCV13 serotypes caused 79% (54/68) of laboratory-confirmed pneumococcal meningitis and 83% (29/35) of culture-confirmed pneumococcal pneumonia. Serotype 1 predominated in meningitis (n = 33) but not in pneumonia patients (n = 1). The incidence of pneumococcal disease was 7.5 per 100,000 among children <5 years of age and 14.8 in persons 5 years of age and above in the study area. When considering CXR-confirmed and blood PCR-positive pneumonia cases as likely pneumococcal, incidence estimates increased to 43.7 and 66.0 per 100,000 in each of these age groups, respectively. Incidence was at least 3-fold higher when we restricted the analysis to the urban area immediately around the study hospitals.

**Conclusions:**

Our findings highlight the important role of S. pneumoniae as a meningitis and pneumonia-causing pathogen in the African meningitis belt. Pneumococcal disease incidence in our population was substantially lower than expected from global models; we hypothesize that poor access to hospital care led us to substantially underestimate the burden of disease. Surveillance is ongoing and will enable an evaluation of PCV impact, providing novel, high quality data from the region.

## Introduction

*Streptococcus pneumoniae* (Sp or pneumococcus) has been the leading cause of pediatric bacterial meningitis and severe pneumonia since the introduction of *Haemophilus influenzae* type b into routine immunization programs [1]. The burden of disease is greatest in low-income countries with high infant and child mortality. In randomized controlled trials, pneumococcal conjugate vaccines (PCVs) were found to be safe and efficacious against invasive pneumococcal disease (IPD), pneumonia with WHO-defined alveolar consolidation or pleural effusion (CXR-confirmed pneumonia), and death in young children [2–5]. Further, post-introduction studies have shown that these vaccines prevent IPD and pneumonia hospitalizations in both immunized and unimmunized age groups [6–8]. The latter are protected through reductions in nasopharyngeal carriage among vaccinated children, who represent the age group most likely to transmit disease [9,10].

The African meningitis belt, first described by Lapeyssonie in the 1960s [11], is characterized by hyperendemic bacterial meningitis during the dry season (approximately December through May), sporadic localized epidemics and large-scale epidemics every 4–10 years [12]. Pneumococci are responsible for the majority of bacterial meningitis cases outside epidemics and contribute to the pronounced meningitis seasonality [13,14]. Pneumococcal meningitis affects all age groups, with incidence rates constant across the age range; and is highly lethal, with case fatality ratios of nearly 50% in patients treated at health facilities [15–17]. A variety of pneumococcal serotypes are seen in children less than 5 years of age, while serotype 1 dominates in older children and adults [13]. In contrast to high income countries, the prevalence of pneumococcal carriage has been found to be high up to adult age [13]. While pneumococcal meningitis epidemiology has been well described in this region, little is known of the burden and etiology of other invasive pneumococcal disease syndromes, particularly pneumonia. Pneumonia appears to be a climate-dependant disease [18–21] and Sp is the most common cause of pneumonia hospitalizations and deaths in the elderly in developed countries [22]. It therefore may also play an important role in both young and older adults in the meningitis belt.

In this study, we aimed to describe the epidemiology of pneumococcal pneumonia and meningitis among children and adults in northern Togo, in the African meningitis belt, before the introduction of pneumococcal conjugate vaccine. We implemented systematic hospital-based surveillance and characterized the incidence, severity and etiology of disease based on clinical, radiological, and laboratory confirmation of cases.

## Methods

### Study area and calendar period

This study was conducted in the Tone and Cinkasse districts of northern Togo, a 900 km^2^ area bordering Burkina Faso to the north and Ghana to the west. The study area had an estimated population of 358,568 (18% under 5 years of age, 4% under 1 year of age) (INSEED Togo) in 2010, increasing at a 3% annual rate. We implemented surveillance at all six health facilities that provide inpatient care for the two districts (Centre Hospitalier Regional in Dapaong (CHR-D), Yendube pediatric mission hospital, Centre Medical des Armees, Polyclinique-Dapaong and Win’Pang private clinic, all located in Dapaong, and the Centre Hospitalier Prefectoral in Cinkasse). Primary health care centers routinely refer severely ill patients to one of these facilities for hospitalization. We enrolled patients of all ages presenting with clinically suspected meningitis [23] or pneumonia (tachypnea for age or difficulty breathing, see case definitions below), who were admitted to hospital, resided in one of the districts and consented to participate in the study. We did not apply any exclusion criteria. Patients with repeat admissions could be enrolled in the study as many times as they were eligible and were assigned a new participant ID at each inclusion. Patients with both pneumonia and meningitis were enrolled in either the meningitis or the pneumonia group depending on their primary cause of hospitalization. The study covered the costs of hospitalization and treatment for all participants. Here, we report on data for the first three years of surveillance, from May 1^st^ 2010 through April 30^th^ 2013. Hib conjugate vaccine was introduced in Togo in 2008, while PCV and meningococcal conjugate vaccine were not in use during the study period.

### Clinical data, chest X-rays and biological specimens

Standardized case-report forms were used to collect demographic and clinical data from all patients, including medical history, signs and symptoms on admission, treatment, and disease outcome. During routine hospital care, pneumonia and meningitis patients are presumptively treated with ceftriaxone, unless culture identifies a drug-resistant pathogen, and oxygen is available to ventilate hypoxic patients. For our study, meningitis patients had cerebro-spinal fluid collected in two tubes, one for routine bacteriological testing and one for PCR. Pneumonia patients had a chest radiograph taken upon admission. X-rays were digitized and read by a pediatrician and a radiologist trained to apply the WHO guidelines for pediatric chest X-ray interpretation [24]. A second pediatrician arbitrated in case of discordance between readers. Pneumonia patients also had two blood draws, based on guidelines of the French Society for Microbiology. The first blood draw was done immediately at the time of enrollment, the second one 30–60 minutes later, following initiation of antibiotic treatment. Blood was inoculated into aerobic and anaerobic hemoculture bottles (for each of the two blood draws: 10 mL in FN and SA bottles for adults, 6 mL in PF bottle for children) for use in the BacT/ALERT semi-automated system (BioMerieux, 69280 Marcy l'Etoile, France), EDTA tubes for whole blood PCR (both blood draws) and serum separator tubes for C-reactive protein evaluation (first blood draw only). CSF and blood were transported from the clinical site to the laboratory by the project courier within 2 hours of collection, with the exception of CSF specimens from the CHP-Cinkasse or from overnight admissions to Yendube hospital, which were inoculated into Trans-Isolate medium and transported to CHR-D as soon as possible.

### Laboratory testing

All CSF specimens and BacT/ALERT-positive blood cultures were sub-cultured on sheep blood agar and chocolate agar plates and processed according to standard bacteriology techniques. CSF also underwent cytology, Gram stain and latex agglutination testing (Pastorex, Biorad, France) at CHR-D and conventional multiplex PCR for detection of *S*. *pneumoniae* (lytA gene), *H*. *influenzae* (bexA gene) and *N*. *meningitidis* (crgA gene) at the Centre Muraz laboratory in Bobo-Dioulasso, Burkina Faso. PCR-positive CSF specimens were serotyped (40 Sp serotypes; Hi type b vs. Hi non-b) or genogrouped (serogroups A, B, C, X, Y, W) according to algorithms published elsewhere [25,26]. Pneumococcal isolates from CSF and blood had antimicrobial susceptibility testing by E-test at CHR-D, were serotyped by PCR at Centre Muraz and subsequently sent to the German National Reference Laboratory for *Streptococci* in Aachen, Germany for confirmatory testing and Quellung. In addition, a random sample of CSF specimens was sent to the WHO Collaborating Center for bacterial meningitis at Institut Pasteur, Paris, France for quality control testing by PCR. Meningitis cases with discrepant culture, latex and/or PCR results had their final etiology determined by the study team after considering quality control and reference laboratory findings: precedence was given to reference laboratory results; in case of discordance between reference laboratories, we examined all results together to determine the most likely etiology. Similarly, Quellung held precedent over PCR-based serotyping for pneumococcal isolates with discrepant findings.

Whole blood was tested by real-time multiplex PCR at the Fondation Merieux Emerging Pathogens Laboratory in Lyon, France. In brief, nucleic acids were extracted from 200 ul of whole blood with the QIAamp DNA blood minikit (Qiagen, Netherlands) and eluted in 100 ul of elution buffer. Five microliters of extracted DNA was then tested with a triplex rtPCR for pneumococcus (lytA), *Haemophilus influenzae* type b, and *Staphylococcus aureus* using the iQ Multiplex Powermix (Bio-Rad, USA). LytA-positive whole blood specimens were further serotyped by multiplex PCR [27]. Finally, serum C reactive-protein (CRP) was measured locally by spectrophotometer.

### Case definitions

We applied the following case definitions in the analysis.

#### Meningitis

Suspected meningitis: clinical diagnosis of meningitis according to the admitting clinician.

Probable bacterial meningitis: suspected meningitis and (visually purulent CSF or white blood cell count ≥ 100/mm3).

Confirmed pneumococcal meningitis: suspected meningitis with *S pneumoniae* identified from CSF by culture or latex agglutination or PCR.

#### Pneumonia

Clinical pneumonia: tachypnea (RR≥60 in patients <2 months of age, RR≥50 in 2–11 month olds, RR≥40 in 1–4 year olds and RR≥20 in patients 5+) or dyspnea

Severe pneumonia: clinical pneumonia with one or more of the following signs lower chest wall indrawing, hypoxia (oxygen saturation <90%), cyanosis, convulsions, prostration or lethargy.

Chest X-ray (CXR) confirmed pneumonia: clinical pneumonia with lobar consolidation or pleural effusion according to standardized radiographic interpretation criteria reported above.

Pneumococcal pneumonia: clinical pneumonia with blood real-time PCR or blood culture positive for pneumococcus.

### Data management and analysis

Data were entered into an EpiInfo 3.5 database and cleaned on a monthly basis. Data analysis was conducted quarterly using Stata 12.0 (StataCorp, College Station, Texas). We described clinical features of study patients including disease severity, treatment and outcome, calculating mean and standard deviation or median and inter-quartile range for continuous variables and proportions for categorical variables. We used t-tests for comparison of means and chi-square tests for proportions, with p = 0.05 as the cut-off for statistical significance. We calculated incidence and case-fatality ratios for pneumonia and meningitis endpoints and investigated the serotype distribution and antimicrobial resistance patterns of pneumococcal cases, by age group, time period (month or year) and geographic location of residence (urban vs. rural).

### Ethics statement

This study was approved by the Togolese national Ethical Review Committee (Comité de Bioéthique pour la Recherche en Santé) and the French data privacy regulator (Commission National Informatique et Libertés). It was also granted a positive opinion by a French Institutional Review Board (Comité de Protection des Personnes de Saint Germain en Laye). All participants provided written informed consent before enrollment into the study.

## Results

### Patient enrollment and clinical characteristics

From May 1^st^ 2010 to April 30^th^ 2013, we enrolled 404 patients with meningitis (104 age <5 years, 25.7%) and 1550 patients with pneumonia (251 age <5 years, 16.2%). An additional 6 meningitis patients and 57 pneumonia patients were eligible for enrollment in the study but did not provide informed consent to participate. We have no further data on these patients. Tables 1 and 2 show demographic and clinical characteristics of study participants.

**Table 1:** Descriptive characteristics of patients with clinical pneumonia.

Total patients	1550	
Median days from symptom onset to admission (IQR)	5 (3–8)	
Median days of hospitalization (IQR)	4 (3–6)	
Median age in years (IQR)	26 (10–41)	
Age group	N	%
<1 year	76	5%
1-4years	175	11%
5-14years	236	15%
15–29 years	389	25%
30–49 years	436	28%
≥50 years	238	15%
Female sex	676	44%
Prior antibiotic treatment	382	25%
Amoxicillin	183	12%
Ceftriaxone	86	6%
Other	149	10%
Severe pneumonia	410	26%
Death during hospitalization	62	4%
Among severe pneumonia cases	41	10%
Among non-severe pneumonia cases	21	2%

**Table 2:** Descriptive characteristics of patients with clinical meningitis.

Total patients	404	
Median days from symptom onset to admission (IQR)	2 (1–4)	
Median days of hospitalization (IQR)	7 (4–10)	
Mean temperature at admission (SD)	38.2 (1.14)	
Median age in years (IQR)	13 (4–31.5)	
Age group		
<1 year	54	13%
1–4 years	50	12%
5–14 years	106	26%
15–29 years	83	21%
30–49 years	80	20%
≥50 years	31	8%
Female sex	196	49%
Prior antibiotic treatment	147	36%
Amoxicillin	55	14%
Chloramphenicol	1	0%
Ceftriaxone	68	17%
Other	62	15%
Sequelae during hospitalization	54	13%
Death during hospitalization	70	17%
<5 years old	16	4%
≥5 years old	54	13%

### Meningitis etiology and incidence

Of the 404 meningitis patients, 383 (94.8%) had a lumbar puncture done. Over half (211, 55.1%) had probable bacterial meningitis, a proportion that differed between patients <5 and ≥5 years of age (41/100 (41.0%) vs. 170/283 (60.1%), p<0.01). Culture, latex agglutination and PCR were performed for 383, 322 and 375 patients, respectively. In total, 132 (34.5%) had an etiology identified including 78 Sp (58.7% of positives). The case-fatality ratio for Sp meningitis was 34.6% and did not vary with age. There was no meningococcal meningitis outbreak during the study period (data not shown).

**Fig 1:**
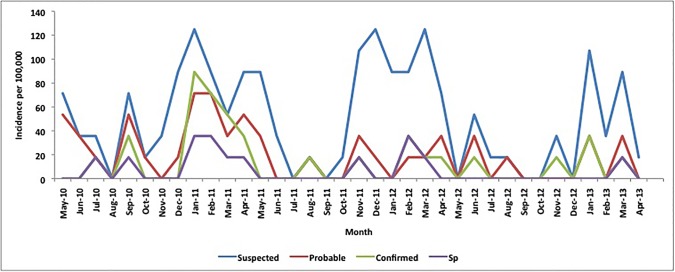
Monthly incidence of suspected, probable, confirmed bacterial and confirmed pneumococcal meningitis incidence per 100,000 in children <5 years of age, northern Togo, May 2010-April 2013. * Note: total number of suspected meningitis cases was 104 over the 3-year period; a single case occurring in a given month equates with a monthly incidence of 17.8 per 100,000.

**Fig 2:**
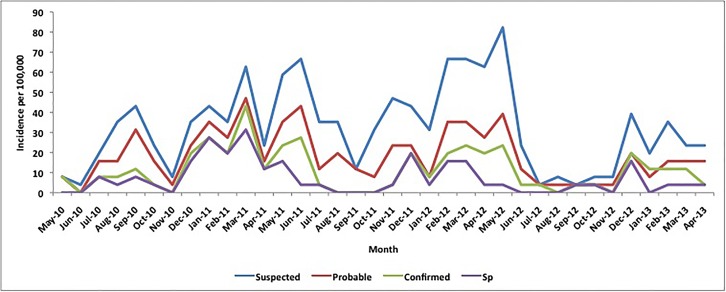
Monthly incidence of suspected, probable, confirmed bacterial and confirmed pneumococcal meningitis incidence per 100,000 in persons ≥5 years of age, northern Togo, May 2010-April 2013. * Note: total number of suspected meningitis cases was 300 over the 3-year period; a single case occurring in a given month equates with a monthly incidence of 4 per 100,000.

The annual incidences of suspected, probable and confirmed Sp meningitis were 36.1, 18.8 and 7.0 per 100,000 over the study period, respectively, with monthly incidences ranging from 3.2 to 77.1, 3.2–45.0 and 0–28.9 per 100,000 and peaking during the dry season each year (Figs 1 and 2). Sp meningitis incidence varied from year to year (2.9–11.3 per 100,000) and was higher in infants (17.9/100,000) than in persons ≥1 year of age (6.5/100,000; p = 0.02). We identified a serotype for 68 pneumococcal meningitis cases; an additional 6 were non-typeable by PCR, 2 were negative by culture and did not have sufficient CSF remaining for serotyping by PCR and 2 were positive by latex agglutination only (Table 3). In children <5 years of age, 12 of 12 serotyped cases were PCV13 vaccine-type (100%), 1 of which was due to serotype 1 (8.3%) whereas in persons ≥5 years of age, 42 of 56 cases were vaccine-type (75.0%) and 32 were serotype 1 (57.1%). The number of serotype 1 cases dropped from 23 in year 1 to 9 in year 2 and 1 in year 3 of the project, whereas the number of non-serotype 1 cases was more stable over time with 18, 13 and 10 cases, respectively. All CSF pneumococcal isolates were sensitive to ceftriaxone and penicillin; 37 (69.8%) were resistant to cotrimoxazole.

**Table 3:** Pneumococcal meningitis serotypes by age group based on PCR or Quellung testing.

Serotype	<5 years of age	≥5 years of age
1	1	32
5	2	4
23F	3	2
12F	0	3
3	0	2
38/25F/A	0	3
10A	0	2
14	2	
19A	1	1
38	0	2
7F/A	2	0
20	0	1
25F	0	1
35F	0	1
6B	1	0
7F	0	1
NT	0	6

### Pneumonia: laboratory and radiological findings

Of the 1550 pneumonia patients enrolled, 1539 (99.2%) had blood collected and 1478 (95.3%) had two blood draws (time between blood draws: median 39 min, IQR 33–45). Bacteria were isolated from blood in 96 patients (6.2%) and included 73 *S*. *pneumoniae* (4.7%), 7 *Salmonella spp*., 6 *S*. *typhi*, 6 *E*. *coli*, 2 *Enterococcus spp*., 1 *H*. *parainfluenzae* and 1 *K*. *pneumoniae*. The proportion of pneumonia cases with pneumococcal bacteremia increased with age, from 0% (0/76) in infants to 8.0% (19/238) in adults ≥50 years of age (p<0.01 for trend). Among 1514 pneumonia patients with real-time PCR testing on whole blood, 124 had Sp identified (8.2%). Among 511 patients with a blood culture and PCR result, 36 of 72 (50.0%) with Sp bacteremia were positive by real-time PCR compared with 88 of 1439 (6.1%) patients without bacteremia (p<0.01); in children age <5 years, these numbers were 0/2 (0%) and 16/223 (7.2%), respectively (p = 0.69). The kappa statistic for agreement between blood culture and real-time PCR for pneumococcal detection was only 0.32. Thus, pneumococcal yield from culture and PCR combined was 2 times higher overall and 8 times higher in children <5 than from culture alone.

The most common serotypes among 35 Sp isolates that could be recovered for testing by PCR and Quellung were serotypes 4 (n = 8), 3 (n = 5) and 19A (n = 5); 29 (83%) were vaccine-type. Comparatively, serotyping of 124 whole blood specimens by PCR identified serotypes 3 (n = 16), 5 (n = 12) and 9V (n = 9) most frequently, with occasional mixed serotypes and 50 cases of indeterminate serotype. Serotypes identified from isolates and whole blood were concordant for all but one patient (Table 4). Pneumococci isolated from blood had very low resistance to ceftriaxone (n = 1, 1.4%) and penicillin (n = 2, 2.7%) but high resistance to cotrimoxazole (n = 53, 72.6%).

**Table 4:** Pneumococcal pneumonia serotypes from blood culture and whole blood PCR.

		Blood isolate serotype											
		Not done	4	19A	3	5	12F	14	38	23F	9V	1	18A
Whole blood PCR serotype	Not done		6	3	2		2	1	2		1		1
	NT	47	1	1	1								
	4	3	1										
	19A	4		1									
	3	14^c^			2^a^								
	5	9^c^ ^d^				3							
	12F	4^b^					1						
	14	1						1					
	38												
	23F									2			
	9V	8^c^									1		
	1	7										1	
	18A												
	19F	4^c^											
	35F	3											
	18	2											
	21	1											
	34	1											
	15B/C	1											
	22F	1											
	6A/B/C	1											
	16F	1^d^											
	20				1^a^								
	33F	1^b^											

Serotypes are ranked in order of their frequency among blood culture isolates. Mixed serotypes appear twice in the table so the total number of results in the table is greater than the total number of patients with a serotype identified. In sum, results from whole blood yielded 60 VT, 2 mixed VT/NVT, 12 NVT and 50 non-typeable by PCR.

^a ^mixed serotypes 3/20 on whole blood

^b^ mixed serotypes 12F/33F on whole blood

^c^: mixed serotypes 3/9V, 5/9V, 19F/9V on whole blood

^d^: mixed serotypes 5/16F on whole blood

In total, 1466 chest X-rays were digitized, read and arbitrated according to the WHO standard methodology: 574 (39.1%) patients had CXR-confirmed pneumonia, 875 did not and 17 (1.2%) had an uninterpretable film. Beyond infancy, the prevalence of CXR-confirmed pneumonia increased with age, from 25.8% in 1–4 year olds to 61.1% in adults ≥50 years of age (p<0.01 for trend). Patients with CXR-confirmed pneumonia were more likely to have severe pneumonia (33.3% vs. 21.1%, p<0.01), to have pneumococcal bacteremia (11.0% vs. 0.5%, p<0.01) and to have CRP≥40 mg/L (86.4% vs. 44.6%, p<0.01) than those without.

### Pneumonia incidence

**Fig 3:**
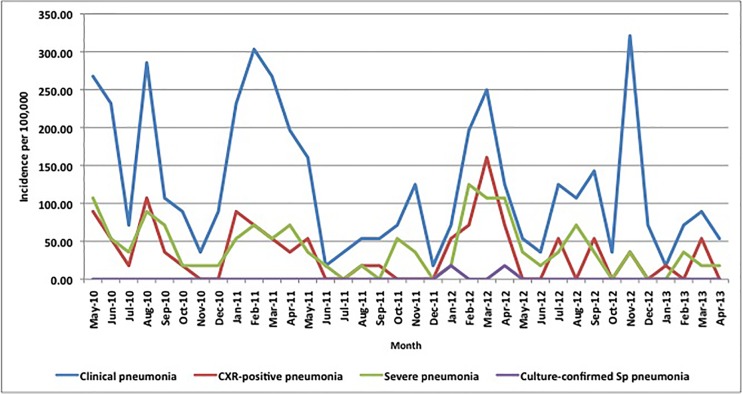
Monthly incidence of clinical, severe, chest X-ray-confirmed and culture-confirmed pneumococcal pneumonia per 100,000 in children <5 years of age in northern Togo, May 2010-April 2013. * Note: total number of clinical pneumonia was 251 over the 3-year period; a single case occurring in a given month equates with a monthly incidence of 17.8 per 100,000.

**Fig 4:**
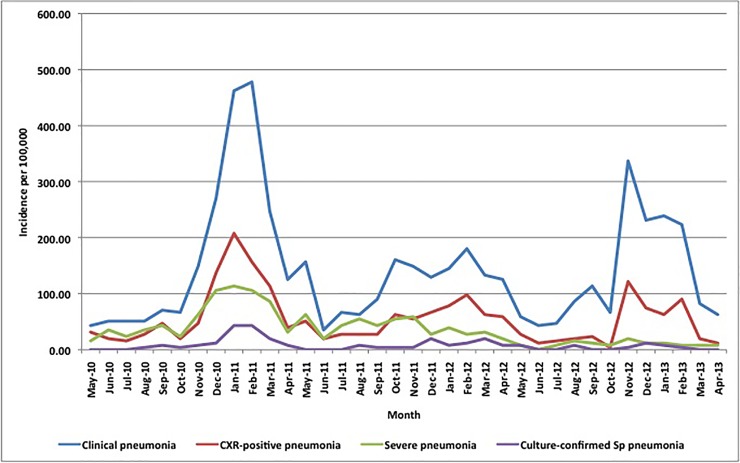
Monthly incidence of clinical, severe, chest X-ray-confirmed and culture-confirmed pneumococcal pneumonia per 100,000 in persons ≥5 years of age in northern Togo, May 2010-April 2013. * Note: total number of clinical pneumonia was 1299 over the 3-year period; a single case occurring in a given month equates with a monthly incidence of 4 per 100,000.

**Fig 5:**
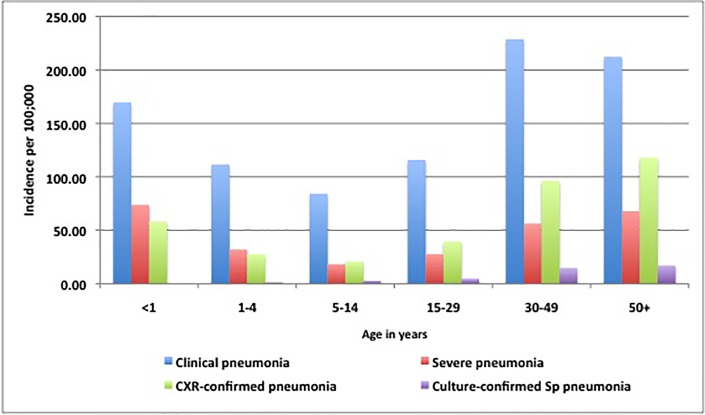
Age-specific incidence of clinical, severe, chest X-ray-confirmed and culture-confirmed pneumococcal pneumonia per 100,000 in northern Togo, May 2010-April 2013.

The incidences of clinical, CXR-confirmed and culture-confirmed Sp pneumonia were 138.3, 51.2 and 6.5 per 100,000 over the project period, respectively (Figs 3 and 4). Pneumonia peaked during the dry season in persons ≥5 years of age but had no clear seasonal pattern in children <5. Clinical and CXR-confirmed pneumonia incidences were highest in adults ≥30 years of age followed by infants <1 year of age and lowest in children 5–14 years old. In contrast, culture-confirmed Sp pneumonia incidences increased steadily with age (Fig 5).

The observed annual incidence of clinical pneumonia was significantly higher in the town of Dapaong than in the rest of the study area, with estimates of 546.9 vs. 63.0 per 100,000 among children age <5 years and 778 vs. 46.3 per 100,000 among persons age 5+ years (p<0.01 for both comparisons). Among children age <5 years, clinical pneumonia incidence remained stable over time in Dapaong but decreased 8-fold over the study period outside Dapaong; in older children and adults, a sharp decline was observed in both the urban and rural areas, with 3.3-fold and 2-fold reductions, respectively. The ratio of urban to rural incidence was also high for CXR-confirmed pneumonia and culture-confirmed pneumococcal pneumonia (Table 2). For each of these endpoints, disease incidence decreased over time in both age groups in the urban and rural areas.

### Incidence of pneumococcal disease

The incidence of laboratory-confirmed pneumococcal disease was 7.5 per 100,000 among children <5 years of age and 14.8 in persons 5 years of age and above in the study area. When considering CXR-confirmed and blood PCR-positive pneumonia cases as likely pneumococcal, incidence estimates increased to 43.7 and 66.0 per 100,000 in each age group, respectively; these numbers were 154.5 and 265.3 per 100,000 after restricting to the analysis to the town of Dapaong.

## Discussion

This study describes the epidemiology of meningitis and pneumonia in all age groups in the African meningitis belt, using state-of-the-art diagnostic methods and a population-based approach. We calculated the incidence of pneumococcal disease by age group, over time and for both urban and rural settings. For children under 5, our estimates of incidence ranged from 7.5 to 154.5 per 100,000, depending on the sensitivity of the case definition chosen and the geographic area considered; for persons 5 years of age and above, the range was 14.8 to 265.3 per 100,000. Comparatively, WHO estimates the incidence of pneumococcal disease in Africa at 3,687 per 100,000 children under 5 for the year 2000; this is more than 20-fold higher than our maximum estimate. The low rates of pneumococcal disease we observed reflect very low rates of hospitalized pneumonia i.e. 124 per 100,000 as compared to the meta-estimate of 2,200 per 100,000 derived from 14 hospitalized pneumonia surveillance studies in Africa (incidence range: 800–10,000 per 100,000) [28]. Reported under-5 mortality in the Savanes region where our study was conducted was 107 per 1,000 live births over the study period [29], so the expected number of under-5 deaths for our study area was approximately 1,500 per year, of which an estimated 300 would be attributable to pneumonia [30]. Only 3 in-hospital pneumonia deaths were observed each year in our study, so if WHO estimates are accurate, this suggests that 99% of fatal pneumonia cases occurred out of hospital. In contrast, UNICEF estimated that in 2013–14, approximately 40% of rural and 40% of lowest wealth quintile children with pneumonia were taken to a health care provider [31]. Several explanations exist for the discrepancy including inaccurate estimates of population size, under-5 mortality, access to care for pneumonia, or the fraction of deaths attributable to pneumonia. Investigating reasons for the enormous gap between expected and observed hospital pneumonia deaths should be a public health priority for Togo, and meningitis belt countries in general, where few data such as ours exist.

As with children, the incidence of adult hospitalized pneumonia in our population also appeared low compared to rural African settings, though to our knowledge evidence from the region is limited to Kenya [32,33] and the low HIV prevalence in Togo could explain its lower disease burden [29]. Because the project covered the cost of hospitalization and treatment for all enrolled patients, we consider that direct medical costs were unlikely to limit access to care in our study. However, disease rates were 8-fold higher in children <5 and 16-fold higher in persons ≥5 in the town of Dapaong than outside Dapaong, suggesting that distance to hospital was a major impediment to accessing care, as seen elsewhere [34]. The decline in incidence observed over the project period is another partial explanation for our low overall rates. This decline may be due to a community health worker program implemented in the region in 2012, which included regular home visits for all children <5 and amoxicillin treatment for those with respiratory infections [35]. Alternately, though we used a sensitive case definition for identification of pneumonia cases and trained surveillance staff to apply this definition systematically, it is possible that clinicians were more likely to categorize patients as malaria or malnutrition than pneumonia, leading to decreased enrollment into the study [36]. Finally, the drop in serotype 1 incidence observed among laboratory-confirmed cases over time may have contributed to the broad declines in all-cause pneumonia.

Our data on meningitis confirm earlier findings from the region, with a stable incidence of disease beyond infancy, annual peaks during the dry season, and a predominance of *S*. *pneumoniae* in the absence of a large meningococcal outbreak [15–17]. Pneumococcus was the leading confirmed meningitis etiology over the three-year period and occurred at similar rates as in earlier studies. Over three-quarters of pneumococcal meningitis cases were preventable by PCV13. Serotype 1 was uncommon in young children and represented >50% of cases in older children and adults. The incidence of serotype 1 Sp fluctuated over time, with a decline in Sp1 coinciding with an increase in other pathogens. Similar waves of serotype 1 meningitis have been described in Ghana [14,37]. As observed previously, over one-third of Sp meningitis cases died in hospital despite receiving appropriate treatment with ceftriaxone; this highlights the importance of disease prevention to reduce morbidity and mortality.

The prevalence of CXR-confirmed pneumonia and of pneumococcal bacteremia suggests that the pneumococcus plays an important role in pneumonia etiology across the age range. The prevalence of pneumococcal bacteremia among pneumonia patients increased with age, from <1% in children under 5 to 8% in adults ≥50 years of age. Despite the implementation of strict procedures for blood culture collection, transport and testing, the low blood volumes obtained from sick children and frequency of prior antimicrobial use likely affected yields [38]. The increase in Sp yield after age 15 years may be due to an increasing specificity of the clinical diagnosis of pneumonia, which is less likely to be confused with other illnesses in adults than in children. Real-time PCR on whole blood allowed identification of additional possible Sp cases: in young children, culture and PCR together detected 8 times more cases than culture alone. Recent data from the PERCH study suggest that lytA PCR may have limited specificity (personal communication, MD Knoll): in several PERCH sites, DNA-emia was seen in healthy controls, which could be explained by the transfer of genetic material from the nasopharynx to the blood in colonized children; however, these results run counter to earlier analyses showing high specificity for blood PCR [39,40]. Elsewhere, we reported findings from latent class analysis models combining laboratory and radiological data from our patients (Blake et al., accepted for publication): we showed that standardized CXR interpretation was the best approach to diagnosing Sp pneumonia and estimated that one third of pneumonia cases were attributable to pneumococcus, a proportion that increased with age from 20 to 50% and was consistent with clinical trials and observational data [5]. These results justify our use of CXR-confirmed pneumonia as a proxy for pneumococcal pneumonia in the estimation of disease incidence.

Our study provides novel information on the incidence and characteristics of pneumococcal meningitis and pneumonia among children and adults in the African meningitis belt, before the introduction of pneumococcal conjugate vaccine. Ongoing surveillance will enable a rigorous evaluation of PCV impact, including direct and indirect effects.

## References

[1] O’Brien KL, Wolfson LJ, Watt JP, Henkle E, Deloria-Knoll M, McCall N, et al Burden of disease caused by Streptococcus pneumoniae in children younger than 5 years: global estimates. Lancet. 2009;374: 893–902. 10.1016/S0140-6736(09)61204-6 19748398

[2] Black S, Shinefield H, Fireman B, Lewis E, Ray P, Hansen JR, et al Efficacy, safety and immunogenicity of heptavalent pneumococcal conjugate vaccine in children. Northern California Kaiser Permanente Vaccine Study Center Group. Pediatr Infect J. 2000;19: 187–95. 10.1097/00006454-200003000-00003 10749457

[3] O’Brien KL, Moulton LH, Reid R, Weatherholtz R, Oski J, Brown L, et al Efficacy and safety of seven-valent conjugate pneumococcal vaccine in American Indian children: group randomised trial. Lancet. 2003;362: 355–361. 10.1016/S0140-6736(03)14022-6 12907008

[4] Klugman KP, Madhi SA, Huebner RE, Kohberger R, Mbelle N, Pierce N. A trial of a 9-valent pneumococcal conjugate vaccine in children with and those without HIV infection. NEnglJMed. 2003;349: 1341–1348. 10.1056/NEJMoa035060 14523142

[5] Cutts FT, Zaman SM, Enwere G, Jaffar S, Levine OS, Okoko JB, et al Efficacy of nine-valent pneumococcal conjugate vaccine against pneumonia and invasive pneumococcal disease in The Gambia: randomised, double-blind, placebo-controlled trial. Lancet. 2005;365: 1139–1146. 10.1016/S0140-6736(05)71876-6 15794968

[6] Direct and indirect effects of routine vaccination of children with 7-valent pneumococcal conjugate vaccine on incidence of invasive pneumococcal disease—United States, 1998–2003. MMWR MorbMortalWklyRep. 2005;54: 893–897. 16163262

[7] Griffin MR, Zhu Y, Moore MR, Whitney CG, Grijalva CG. U.S. hospitalizations for pneumonia after a decade of pneumococcal vaccination. N Engl J Med. 369: 155–63. 10.1056/NEJMoa1209165 23841730 PMC4877190

[8] Miller E, Andrews NJ, Waight PA, Slack MP, George RC. Herd immunity and serotype replacement 4 years after seven-valent pneumococcal conjugate vaccination in England and Wales: an observational cohort study. Lancet Infect Dis. 11: 760–8. 10.1016/S1473-3099(11)70090-1 21621466

[9] Millar EV, Watt JP, Bronsdon MA, Dallas J, Reid R, Santosham M, et al Indirect effect of 7-valent pneumococcal conjugate vaccine on pneumococcal colonization among unvaccinated household members. Clin Infect Dis. 2008;47: 989–96. 10.1086/591966 18781875

[10] Givon-Lavi N, Fraser D, Dagan R. Vaccination of day-care center attendees reduces carriage of Streptococcus pneumoniae among their younger siblings. Pediatr Infect J. 2003;22: 524–32. 10.1097/01.inf.0000069760.65826.f2 12799509

[11] Lapeyssonnie L. [Cerebrospinal Meningitis in Africa]. Bull World Health Organ. 1963;28 Suppl: 1–114. PMC2554630 14259333

[12] Mueller JE, Gessner BD. A hypothetical explanatory model for meningococcal meningitis in the African meningitis belt. Int J Infect Dis. 14: e553–9. 10.1016/j.ijid.2009.08.013 20018546

[13] Mueller JE, Yaro S, Ouédraogo MS, Levina N, Njanpop-Lafourcade B-M, Tall H, et al Pneumococci in the African Meningitis Belt: Meningitis Incidence and Carriage Prevalence in Children and Adults. Beall B, editor. PLoS ONE. 2012;7: e52464 10.1371/journal.pone.0052464 23285051 PMC3527509

[14] Leimkugel J, Adams FA, Gagneux S, Pfluger V, Flierl C, Awine E, et al An Outbreak of Serotype 1 Streptococcus pneumoniae Meningitis in Northern Ghana with Features That Are Characteristic of Neisseria meningitidis Meningitis Epidemics. JInfectDis. 2005;192: 192–199. 10.1086/431151 15962213

[15] Yaro S, Lourd M, Traore Y, Njanpop-Lafourcade BM, Sawadogo A, Sangare L, et al Epidemiological and molecular characteristics of a highly lethal pneumococcal meningitis epidemic in Burkina Faso. Clin Infect Dis. 2006;43: 693–700. 10.1086/506940 16912941

[16] Traore Y, Tameklo TA, Njanpop-Lafourcade BM, Lourd M, Yaro S, Niamba D, et al Incidence, seasonality, age distribution, and mortality of pneumococcal meningitis in Burkina Faso and Togo. Clin Infect Dis. 2009;48 Suppl 2: S181–9. 19191614 10.1086/596498

[17] Gessner B. Pneumococcal meningitis in Burkina Faso and Togo is common, seasonal, affects all age groups, and is highly lethal. Clin Infect Dis. 2008;47 (S).

[18] Bunker A, Wildenhain J, Vandenbergh A, Henschke N, Rocklöv J, Hajat S, et al Effects of Air Temperature on Climate-Sensitive Mortality and Morbidity Outcomes in the Elderly; a Systematic Review and Meta-analysis of Epidemiological Evidence. EBioMedicine. 2016;6: 258–268. 10.1016/j.ebiom.2016.02.034 27211569 PMC4856745

[19] Kim J, Kim J-H, Cheong H-K, Kim H, Honda Y, Ha M, et al Effect of Climate Factors on the Childhood Pneumonia in Papua New Guinea: A Time-Series Analysis. Int J Environ Res Public Health. 2016;13: 213 10.3390/ijerph13020213 26891307 PMC4772233

[20] Ben-Shimol S, Greenberg D, Hazan G, Shemer-Avni Y, Givon-Lavi N, Dagan R. Seasonality of both bacteremic and nonbacteremic pneumonia coincides with viral lower respiratory tract infections in early childhood, in contrast to nonpneumonia invasive pneumococcal disease, in the pre-pneumococcal conjugate vaccine era. Clin Infect Dis Off Publ Infect Dis Soc Am. 2015;60: 1384–1387. 10.1093/cid/civ023 25595749

[21] Weinberger DM, Grant LR, Steiner CA, Weatherholtz R, Santosham M, Viboud C, et al Seasonal Drivers of Pneumococcal Disease Incidence: Impact of Bacterial Carriage and Viral Activity. Clin Infect Dis. 2014;58: 188–194. 10.1093/cid/cit721 24190895 PMC3871795

[22] Said MA, Johnson HL, Nonyane BAS, Deloria-Knoll M, O′Brien KL, for the AGEDD Adult Pneumococcal Burden Study Team. Estimating the Burden of Pneumococcal Pneumonia among Adults: A Systematic Review and Meta-Analysis of Diagnostic Techniques. Hill PC, editor. PLoS ONE. 2013;8: e60273 10.1371/journal.pone.0060273 23565216 PMC3615022

[23] WHO: Laboratory methods for the diagnosis of meningitis caused by Neisseria meningitidis, Streptococcus pneumoniae, and Haemophilus influenzae. WHO Communicable disease surveillance and response.; 1999 p.

[24] Cherian T, Mulholland EK, Carlin JB, Ostensen H, Amin R, de Campo M, et al Standardized interpretation of paediatric chest radiographs for the diagnosis of pneumonia in epidemiological studies. BullWorld Health Organ. 2005;83: 353–359. PMC2626240 15976876

[25] Parent du Chatelet I, Traore Y, Gessner BD, Antignac A, Naccro B, Njanpop-Lafourcade BM, et al Bacterial meningitis in Burkina Faso: surveillance using field-based polymerase chain reaction testing. Clin Infect Dis. 2005;40: 17–25. 10.1086/426436 15614687

[26] Njanpop Lafourcade BM, Sanou O, van der Linden M, Levina N, Karanfil M, Yaro S, et al Serotyping pneumococcal meningitis cases in the African meningitis belt by use of multiplex PCR with cerebrospinal fluid. J Clin Microbiol. 48: 612–4. 10.1128/JCM.01402-09 20007384 PMC2815583

[27] Messaoudi M, Milenkov M, Albrich WC, van der Linden MPG, Bénet T, Chou M, et al The Relevance of a Novel Quantitative Assay to Detect up to 40 Major Streptococcus pneumoniae Serotypes Directly in Clinical Nasopharyngeal and Blood Specimens. de Lencastre H, editor. PLOS ONE. 2016;11: e0151428 10.1371/journal.pone.0151428 26986831 PMC4795784

[28] Nair H, Simões EA, Rudan I, Gessner BD, Azziz-Baumgartner E, Zhang JSF, et al Global and regional burden of hospital admissions for severe acute lower respiratory infections in young children in 2010: a systematic analysis. The Lancet. 2013;381: 1380–1390. 10.1016/S0140-6736(12)61901-1 PMC3986472 23369797

[29] Demographic and Health Surveys: Togo 2013 [Internet]. Calverton, MD; 2015. Available: http://www.measuredhs.com/publications/publication-FR101-DHS-Final-Reports.cfm

[30] Johnson HL, Deloria-Knoll M, Levine OS, Stoszek SK, Freimanis Hance L, Reithinger R, et al Systematic evaluation of serotypes causing invasive pneumococcal disease among children under five: the pneumococcal global serotype project. PLoS Med. 7 Available: http://www.ncbi.nlm.nih.gov/entrez/query.fcgi?cmd=Retrieve&db=PubMed&dopt=Citation&list_uids=20957191 10.1371/journal.pmed.1000348 PMC2950132 20957191

[31] State of the World’s Children 2014. UNICEF; 2014.

[32] Tornheim JA, Manya AS, Oyando N, Kabaka S, Breiman RF, Feikin DR. The epidemiology of hospitalized pneumonia in rural Kenya: the potential of surveillance data in setting public health priorities. Int J Infect Dis IJID Off Publ Int Soc Infect Dis. 2007;11: 536–543. 10.1016/j.ijid.2007.03.006 17537660

[33] Etyang AO, Munge K, Bunyasi EW, Matata L, Ndila C, Kapesa S, et al Burden of disease in adults admitted to hospital in a rural region of coastal Kenya: an analysis of data from linked clinical and demographic surveillance systems. Lancet Glob Health. 2014;2: e216–224. 10.1016/S2214-109X(14)70023-3 24782954 PMC3986034

[34] Moisi JC, Nokes DJ, Gatakaa H, Williams TN, Bauni E, Levine OS, et al Sensitivity of hospital-based surveillance for severe disease: a geographic information system analysis of access to care in Kilifi district, Kenya. Bull World Health Organ. 2011;89: 102–11. 10.2471/BLT.10.080796 21346921 PMC3040379

[35] Sazawal S, Black RE, Pneumonia Case Management Trials Group. Effect of pneumonia case management on mortality in neonates, infants, and preschool children: a meta-analysis of community-based trials. Lancet Infect Dis. 2003;3: 547–556. 12954560 10.1016/s1473-3099(03)00737-0

[36] English M, Punt J, Mwangi I, McHugh K, Marsh K. Clinical overlap between malaria and severe pneumonia in Africa children in hospital. Trans R Soc Trop Med Hyg. 1996;90: 658–662. 9015508 10.1016/s0035-9203(96)90423-x

[37] Kwambana-Adams BA, Asiedu-Bekoe F, Sarkodie B, Afreh OK, Kuma GK, Owusu-Okyere G, et al An outbreak of pneumococcal meningitis among older children (≥5 years) and adults after the implementation of an infant vaccination programme with the 13-valent pneumococcal conjugate vaccine in Ghana. BMC Infect Dis. 2016;16. 10.1186/s12879-016-1914-3 PMC5070171 27756235

[38] Isaacman DJ, Karasic RB, Reynolds EA, Kost SI. Effect of number of blood cultures and volume of blood on detection of bacteremia in children. JPediatr. 1996;128: 190–195. 8636810 10.1016/s0022-3476(96)70388-8

[39] Azzari C, Cortimiglia M, Moriondo M, Canessa C, Lippi F, Ghiori F, et al Pneumococcal DNA is not detectable in the blood of healthy carrier children by real-time PCR targeting the lytA gene. J Med Microbiol. 60: 710–4. 10.1099/jmm.0.028357-0 21349984 PMC3167920

[40] Rouphael N, Steyn S, Bangert M, Sampson JS, Adrian P, Madhi SA, et al Use of 2 pneumococcal common protein real-time polymerase chain reaction assays in healthy children colonized with Streptococcus pneumoniae. Diagn Microbiol Infect Dis. 70: 452–4. 10.1016/j.diagmicrobio.2010.09.006 21397430

